# Supplementary material for: Characterization of bZIP Transcription Factors in Transcriptome of Chrysanthemum mongolicum and Roles of CmbZIP9 in Drought Stress Resistance
Source: Plants (Basel). 2024 Jul 26;13(15):2064. doi: 10.3390/plants13152064 (PMC11314283; doi:10.3390/plants13152064)
Supplement: Supplementary file 1 [file plants-13-02064-s001.zip › plants-3112964-supplementary.pdf]

Characterization of bZIP Transcription Factors in Transcriptome of *Chrysanthemum mongolicum* and Roles of *CmbZIP9* in Drought Stress Resistance

Xuan Wang [1], Yuan Meng [1], Shaowei Zhang [1], Zihan Wang [1], Kaimei Zhang [2], Tingting Gao [1] and Yueping Ma [1,*]

[1] College of Life and Health Sciences, Northeastern University, Shenyang 110169, China; wangxuan99000@163.com (X.W.); 2201455@stu.neu.edu.cn (Y.M.); 2201483@stu.neu.edu.cn (S.Z.); 2301526@stu.neu.edu.cn (Z.W.); gaotingting@mail.neu.edu.cn (T.G.)

[2] Co-Innovation Center for Sustainable Forestry in Southern China, College of Life Sciences, Nanjing Forestry University, Nanjing 210037, China; kaimeizhang@njfu.edu.cn

* Correspondence: yuepingma@mail.neu.edu.cn; Tel.: +86-024-83656098

Table S1 Physicochemical properties of *bZIP* genes identified in *C. mongolicum*

Gene ID	Group	CDS	No. of AA	Mw (kDa)	PI	Subcellular localization prediction
Cluster-11194.0	S	588	195	22691.03	5.28	Nucleus cytoplasm
Cluster-19700.3456	A	621	206	34412.65	9.45	Nucleus
Cluster-19700.19587	A	1479	492	41585.39	9.26	Nucleus
Cluster-19700.19744	I	1488	495	60328.07	6.45	Nucleus
Cluster-19700.4505	A	1776	591	33963.29	8.92	Nucleus
Cluster-19700.506	S	1791	596	16511.49	5.6	Nucleus
Cluster-19700.8422	G	1872	623	15600.31	5.76	Nucleus
Cluster-3627.1	S	2100	699	14433.07	9.97	Nucleus
Cluster-16529.0	A	2304	767	27905.93	9.41	Nucleus
Cluster-19700.10400	A	2364	787	44392.61	8.98	Nucleus
Cluster-19700.11462	S	2403	800	18938.08	5.59	Nucleus
Cluster-19474.0	I	3489	1162	47505.93	6.54	Nucleus
Cluster-19700.12208	A	3546	1181	18036.67	10.06	Nucleus
Cluster-19700.20449	I	3726	1241	29307.72	6.51	Nucleus mitochondria chloroplast
Cluster-19700.347	I	3936	1311	36227.84	5.97	Nucleus Cytoplasmic membrane
Cluster-23609.0	S	4161	1386	19135.36	5.21	Nucleus
Cluster-25258.0	S	4173	1390	16092.44	9.25	Nucleus
Cluster-28859.1	A	4239	1412	23655.87	7.9	Nucleus
Cluster-14882.0	S	4437	1478	16765.86	5.41	Nucleus
Cluster-19700.27736	I	4914	1637	35454.73	6.07	Nucleus chloroplast cytoplasm
Cluster-19700.5498	C	5088	1695	47493.77	6.57	Nucleus
Cluster-26546.0	S	5328	1775	17412.48	6.09	Nucleus
Cluster-8953.0	C	5469	1822	19115.96	5.81	Nucleus
Cluster-19700.13551	C	1035	344	38159.77	5.79	nucleus
Cluster-19700.14222	S	423	140	15876.13	8.05	nucleus
Cluster-19700.8714	A	669	222	24580.21	4.92	nucleus cytoplasm
Cluster-19700.23991	I	954	317	34758.4	7.11	nucleus cytoplasm
Cluster-19700.31950	S	486	161	18244.26	5.71	nucleus

Table S2 Characterized the motif in CmbZIPs

Motif	The name of the query motif	Sequence logos of the CMbZIPs motifs
1	ELDLVDERRQKRMJSNRESAARS-RERKQAYLDELENQV	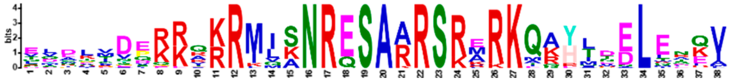
2	LSAQLTLTQQDYVGLESENSVL-KARLZELEQRLQLLBELNE	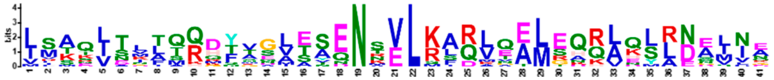
3	LARQGSJYNLTFDELQNTLGDLGKPFGSMN-LDELLKSIWTAEG	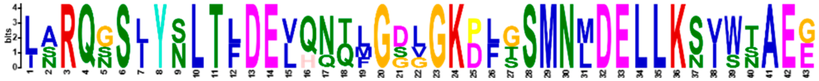
4	RQQTLGEMTLEDFLVKAGVVAE	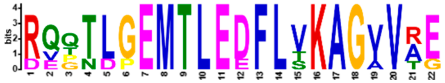
5	TGEIANCSDGYNLGMHQFPYNQPNFFRNQKQQE PPQYHHNHQSNMSNNHH	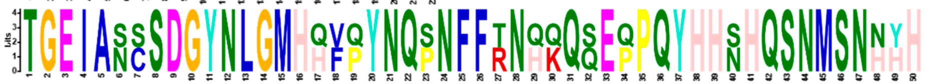
6	KTVDEVWKDI	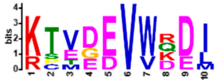
7	KREGIKRSAGGDI-APTTRHYRSVSMDSFMGGMNFAEESPKLPP-SPGGQI	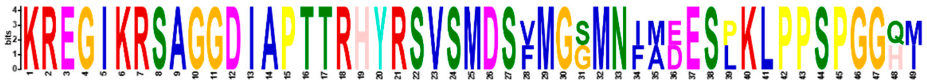
8	YANQPILASADMIQY	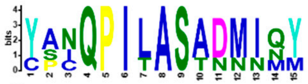
9	RLKEENERLKR	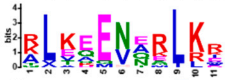
10	SPFRPSHHRRAQSETPFRFPT	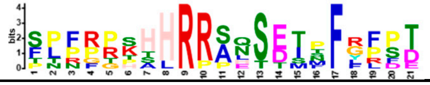

Table S3 Primers used in this study

Primer	Sequence	Application
M551ZIP F	ACTTCTAGAATGGCCGCAAACCCTCTCT	Cloning the *CmbZIP*
M551ZIP R	ACTGGTACCCCAAATGTTACGATGAGAATC	
qZip F	GAATGGGCAACGTCTCTCCA	Expression analysis of *CmbZIP*
qZip R	CCATCACGTGTTGACCTGGA	
NtSOD F	AGCTACATGACGCCATTTCC	Expression analysis of *SOD*
NtSOD R	CTTCAACGTGTTATCAGTAG	
NtAREB F	TCTTCACAGCAAAAGCCTC	Expression analysis of *AREB*
NtAREB R	GTGACCCCATTATGCAATC	
NtLEA14 F	CTCCGTTCCCGTACCTATCA	Expression analysis of *LEA*
NtLEA14 R	CAATCTGCGCCAATATCCTT	
NtSAMDC F	TGCACTCTCAACCATTCACA	Expression analysis of *SAMDC*
NtSAMDC R	GAACTCATCTGGCTCGAAAC	
NtADC F	GCTAAGCTCAGGACCAAGCA	Expression analysis of *ADC*
NtADC R	CACCAACACCATCAGCAAGC	
NtActin F	CATTGTGCTCAGTGGTGG	Expression reference
NtActin R	AAGGGATGCGAGGATGGA	

A

```
1     ATGGCCGCAAACCCTCTCTGGTCTCAAAATCTTACTCCGAAGAATTCTAGTGTCACGATG
1      M  A  A  N  P  L  W  S  Q  N  L  T  P  K  N  S  S  V  T  M
61    ACTATGGATTCCCAGTCGTCGATATGTGCTGATAGTCCAACCTCGGATACCAAACCAAAG
21     T  M  D  S  Q  S  S  I  C  A  D  S  P  T  S  D  T  K  P  K
121   GTTAGGCGGGATAACAATGTGATTGGGGCAACCAGTGATGATGAACAATCAGATGATGAT
41     V  R  R  D  N  N  V  I  G  A  T  S  D  D  E  Q  S  D  D  D
181   GATACGGAGATTGAAGCTGGTCAATGTGAACAGAGCAACGATCAAATGGATGTTAAACGG
61     D  T  E  I  E  A  G  Q  C  E  Q  S  N  D  Q  M  D  V  K  R
241   ATTAAAAGGATGGTTTCAAATAGAGAATCTGCTAGGCGCTCGAGAAAAAGAAAGCAAGCA
81     I  K  R  M  V  S  N  R  E  S  A  R  R  S  R  K  R  K  Q  A
301   CATCTAACAGATCTTGAGCAACAAGTGGAACAACTGCGAGGAGAGTACTCAACTTTGTTC
101    H  L  T  D  L  E  Q  Q  V  E  Q  L  R  G  E  Y  S  T  L  F
361   AAGCAACTAACAAATGCTAGCCAACAGTTTAAAGATGCTTCAACTAACAACCGAGTGCTC
121    K  Q  L  T  N  A  S  Q  Q  F  K  D  A  S  T  N  N  R  V  L
421   AAGTCAGATGTGGAAGCCTTGAGAGCTAAGGTGAAGCTGGCGGAAGATATGGTTGCTAGA
141    K  S  D  V  E  A  L  R  A  K  V  K  L  A  E  D  M  V  A  R
481   GGCTCATTAACATCTAGTCTTAGCCACCTTATTCAGAACCATTTAACCACTCCCCCGATG
161    G  S  L  T  S  S  L  S  H  L  I  Q  N  H  L  T  T  P  P  M
541   TTCAACAGTCAAAACATGTCTCGAATGGGCAACGTCTCTCCAACAATAACTGTTCGTGGG
181    F  N  S  Q  N  M  S  R  M  G  N  V  S  P  T  I  T  V  R  G
601   GATGACCATGGGTCCCACCCTGGACTTTCTGTTCCAGGTCAACACGTGATGGTTGGACTA
201    D  D  H  G  S  H  P  G  L  S  V  P  G  Q  H  V  M  V  G  L
661   GGTAATAATCCCGACATTTTTAACGCTAATGTGAAAAATGGTATAACTAGTGATGCTGGA
221    G  N  N  P  D  I  F  N  A  N  V  K  N  G  I  T  S  D  A  G
721   AGCTGTGTATCTGATATATGGCCTTTGGATTCTCATCGTAACATTTGA
241    S  C  V  S  D  I  W  P  L  D  S  H  R  N  I  *
```

B

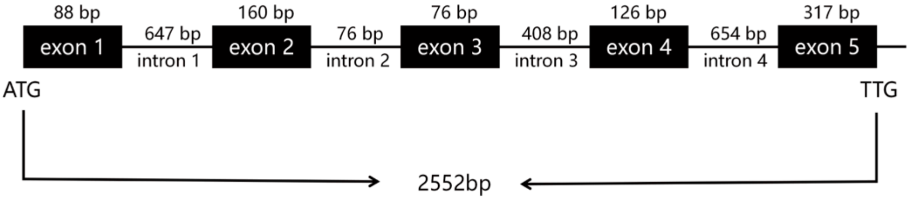

Figure S1. Structure of CmbZIP9 in C. mongolicum. (A) The complete cDNA sequence of CmbZIP9 and its amino acids. The conserved basic region and leucine zipper motif are indicated by dotted and double solid lines, respectively. (B) Structure of the full-length CmbZIP9 gene. Black boxes represent exons and thin lines represent introns. The size of each fragment is shown in numbers. The codons for start (ATG) and stop (TAG) are marked.

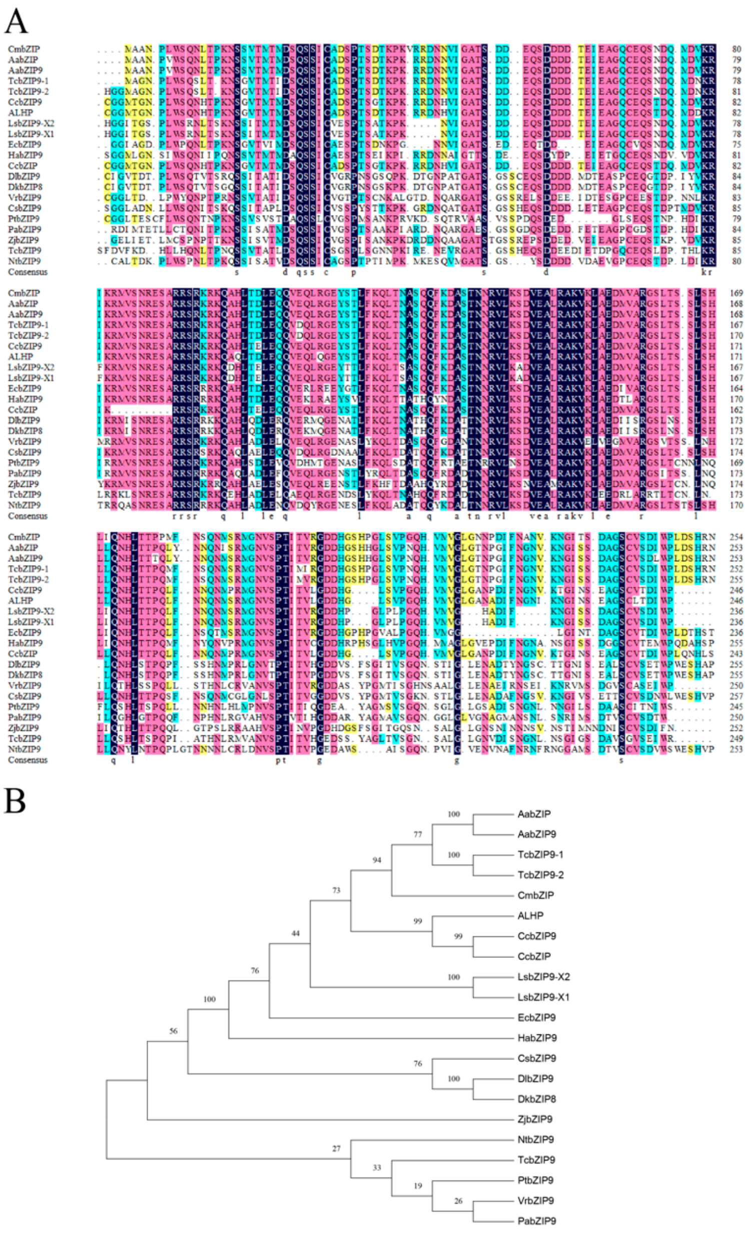

Figure S2. Comparison of CmbZIP with bZIP homologs. (A) Alignment amino acid sequence of bZIP- -related in plant with high sequence similarity to CmbZIP; (B) Phylogeny of bZIP-related proteins. AabZIP and AabZIP9, *Artemisia annua* (PWA82790.1 and QAU20958.1); TcbZIP9, *Tanacetum cinerariifolium* (GEY13937.1) ; LsbZIP9, *Lactuca sativa* (XP_023742342.1) ; HabZIP9, *Helianthus annuus* (XP_022029119.1) ; DkbZIP8, *Diospyros kaki* (AZL19532.1) ; CsbZIP9, *Camellia sinensis* (XP_028078814.1) ; VvbZIP9, *Vitis vinifera* (RVX17682.1); CfbZIP1, *Cephalotus follicularis* (GAV63659.1) ; PabZIP9, *Prunus avium* (XP_021819356.1) ; ZjbZIP9, *Ziziphus jujuba* (XP_015868915.1); CcbZIP, *Cynara cardunculus var. scolymus* (XP_024996183.1); AlbZIP, *Arctium lappa* (KAI3692304.1); EcbZIP9, *Erigeron canadensis* (XP_043629098.1); DlbZIP, *Diospyros lotus* (XP_052209959.1); AabZIP, *Artemisia annua* (QAU20958.1); *Nicotiana tabacum* (XP_016446706.1); CmbZIP9, *C. mongolicum.*

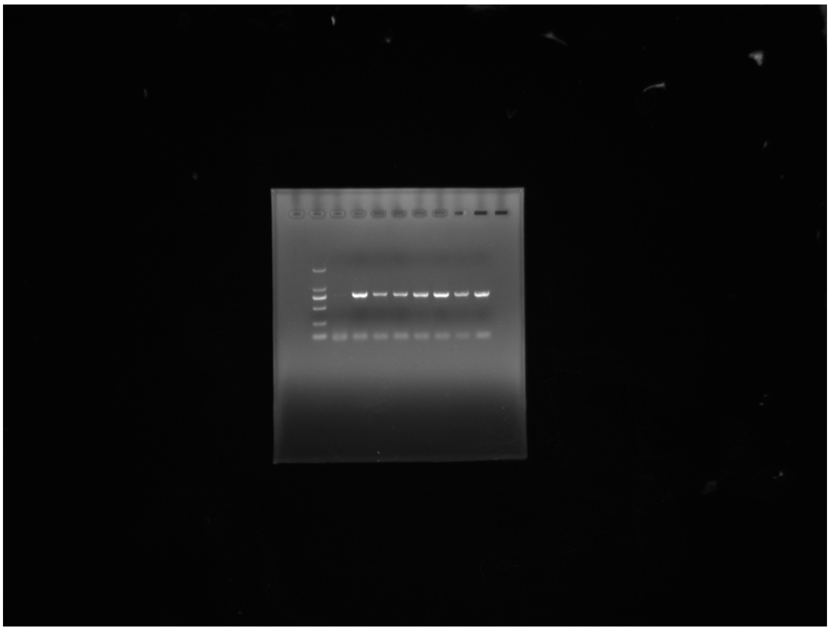

Figure S3. Uncropped gels.
